# Child with Closed Head Injury and Persistent Vomiting

**DOI:** 10.5811/cpcem.4814

**Published:** 2024-04-17

**Authors:** Abdullah Khan

**Affiliations:** Sidra Medical and Research Centre, Department of Emergency Medicine, Doha, Qatar

**Keywords:** closed head injury, rare-earth magnets, vomiting

## Abstract

**Case Presentation:**

We present the case of a six-year-old child with autism who presented with persistent vomiting in the setting of a closed head injury (CHI). Computed tomography of the head was normal, but due to persistent vomiting a radiograph of the abdomen was done, which showed multiple, rare-earth magnets in the abdomen. There was no history of witnessed ingestion. These magnets had caused enteroenteric fistula formation leading to persistent vomiting.

**Discussion:**

In the setting of CHI, vomiting can be a sign of concussion or intracranial hemorrhage. In cases of CHI where intracranial pathology is ruled out and vomiting still persists, it is important to explore intra-abdominal causes of vomiting, especially in developmentally challenged children as they have higher incidence of unwitnessed foreign body ingestions.

Population Health Research Capsule What do we already know about this clinical entity?

*Rare-earth magnets are more powerful than regular magnets, and when ingested in multiple numbers can cause intestinal complications.*

What is the major impact of the image(s)?

*In an autistic child presenting with closed head injury, abdominal imaging incidentally showed ingested rare-earth magnets.*

How might this improve emergency medicine practice?

*Foreign body ingestions should be considered in the differential diagnosis of vomiting. especially when evaluating children with neurodevelopmental disabilities.*

## CASE PRESENTATION

**Image 1.**
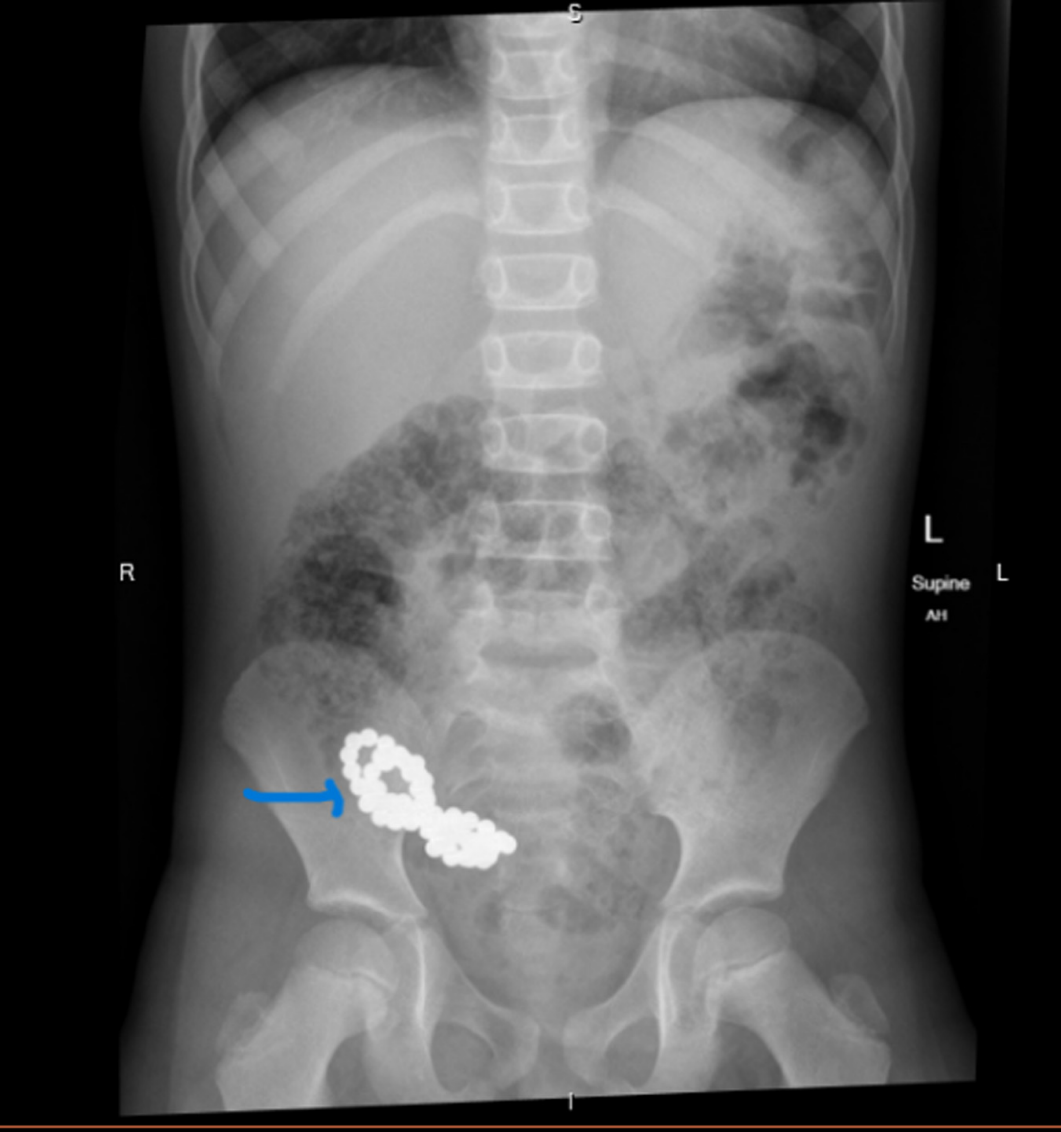
Supine radiograph of the abdomen showing multiple rare-earth magnets (blue arrow).

A six-year-old autistic child presented to the emergency department with multiple episodes of non-bloody, non-bilious vomiting after sustaining a closed head injury (CHI). The patient had fallen face forward on the ground from a height of five to six stairsteps. No associated loss of consciousness, seizures, abdominal pain, difficulty breathing, ear, eye, or nasal discharges were reported. The physical examination revealed a two-centimeter contusion on the forehead. The rest of the ocular, auditory, abdominal, respiratory examinations including Glasgow Coma scale were normal. Initially the patient received ondansetron, but vomiting continued after the medication. Due to persistent vomiting, complete blood count, blood electrolytes, liver function tests, lipase level, and urinalysis were obtained from the laboratory, and CT of the head without contrast was performed. All lab tests and CT were normal. A radiograph of the abdomen incidentally showed a cluster of small round balls of rare-earth magnets with no signs of obstruction or pneumoperitoneum (Image 1).

**Image 2.**
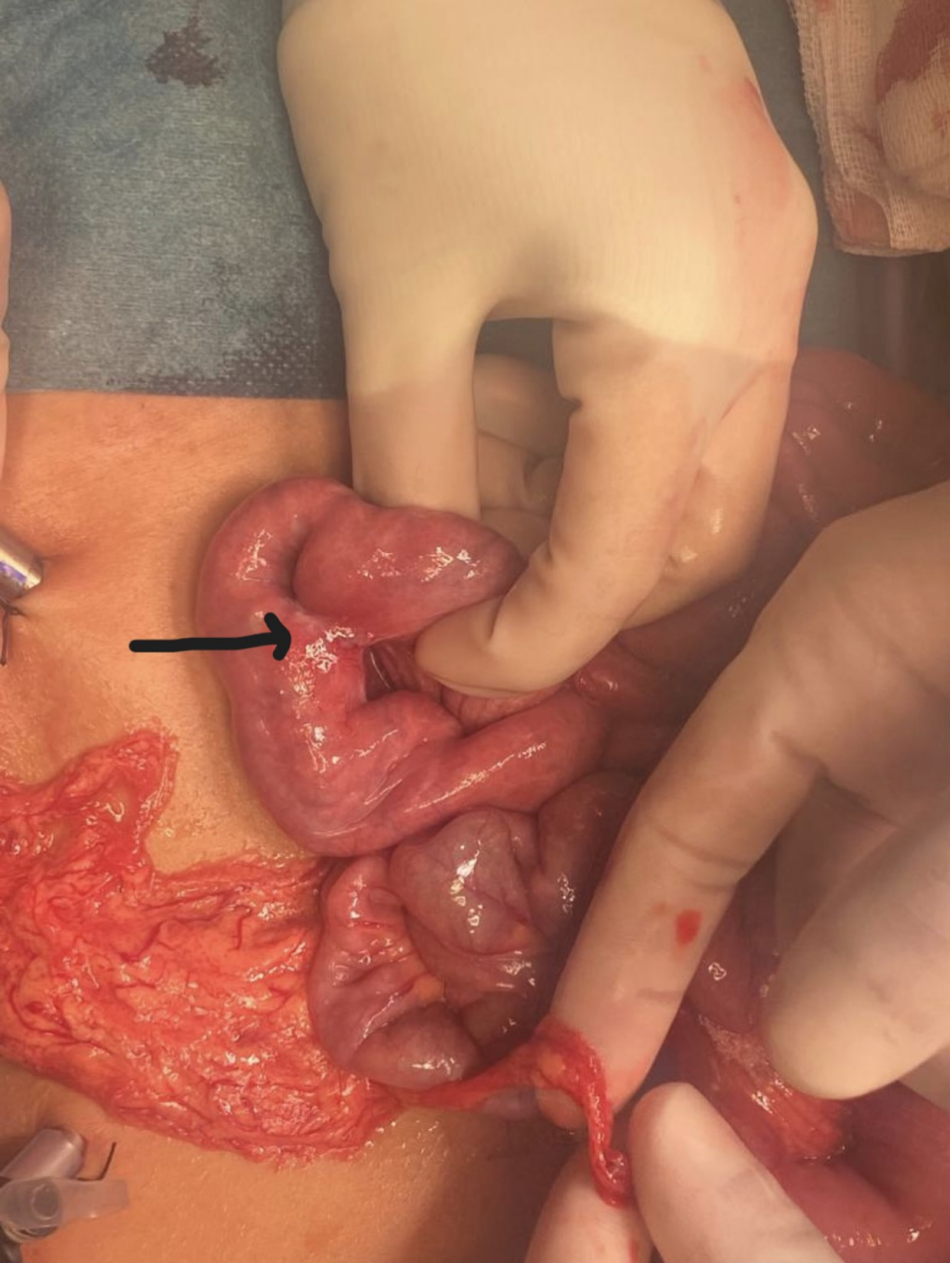
View of bowel during laparotomy showing enteroenteric fistula (black arrow).

**Image 3.**
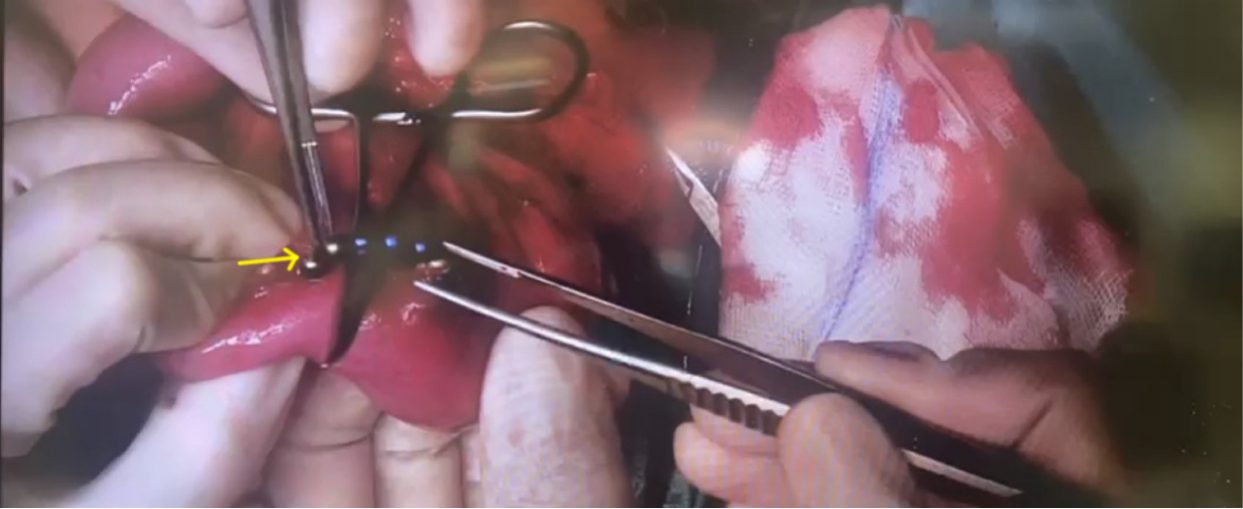
Intraoperatively, fistula is resected, and rare-earth magnets are removed (yellow arrow).

There was no history of witnessed ingestion. General surgery was consulted, and the patient was admitted to the hospital. Initially, the patient was managed conservatively with antiemetics and laxatives; but due to persistent vomiting and lack of movement of the magnets, the patient was taken to the operating room. During the laparotomy, it was noticed that the magnets had caused formation of an enteroenteric fistula (Image 2). The fistula was divided, the magnets were extracted, and the edges of the fistula were closed. (Image 3). The patient recovered without any complications and was discharged from the hospital.

## DISCUSSION

Rare-earth magnets (neodymium magnets) are commercially sold as 3–6 millimeters round recreational objects; they are five to 10 times more powerful than normal magnets. When more than one of these magnets are ingested, the bowel can get compressed between them, which leads to obstruction, necrosis, perforation, and fistula formation.^1^ Due to the small size of these magnets, patients can develop localized intestinal perforations and fistulas without significant symptoms and radiologic findings. Therefore, in patients with ingestion of multiple rare-earth magnets, surgical or endoscopic removal of the magnets should be performed even in asymptomatic patients.^2^ The incidence of foreign body ingestion is higher in toddlers and preschool children, whereas a higher incidence of ingestion is noticed at older age in children with neurological disabilities.^3^ Because foreign body ingestions in these children are often unwitnessed, it presents a challenge in diagnosis.^1^^,^^3^ In summary, foreign body ingestions should be in the differential diagnosis in children with neurological disability presenting with unexplained vomiting. Additionally, in cases with ingestion of multiple rare-earth magnets, conservative management may *not* be the appropriate choice.
